# Supplementary figures and images for: Perception is associated with the brain’s metabolic response to sensory stimulation
Source: eLife. 2022 Feb 28;11:e71016. doi: 10.7554/eLife.71016 (PMC9038191; doi:10.7554/eLife.71016)

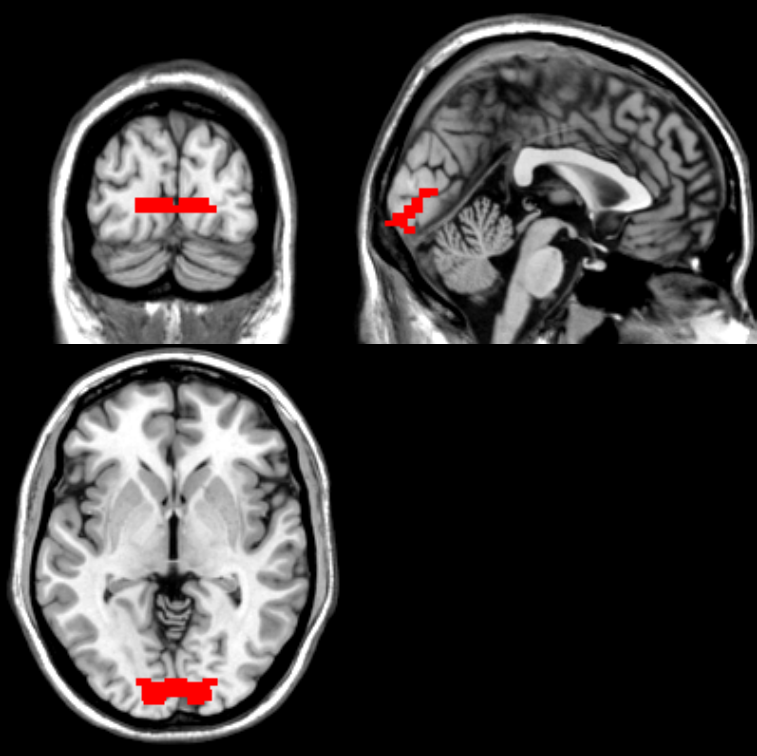

Supplement: Figure 1—source data 2. [file elife-71016-fig1-data2.zip › Figure 1 - Source Data 3/fig_1_data/mni_ba17_d12D_91_39_70.png]

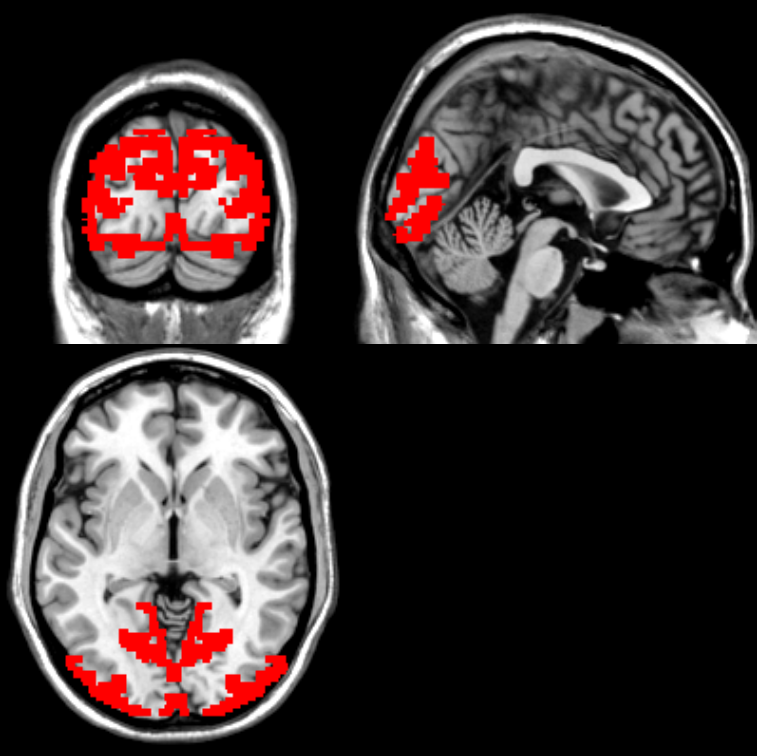

Supplement: Figure 1—source data 2. [file elife-71016-fig1-data2.zip › Figure 1 - Source Data 3/fig_1_data/mni_ba1819_d12D_91_39_70.png]

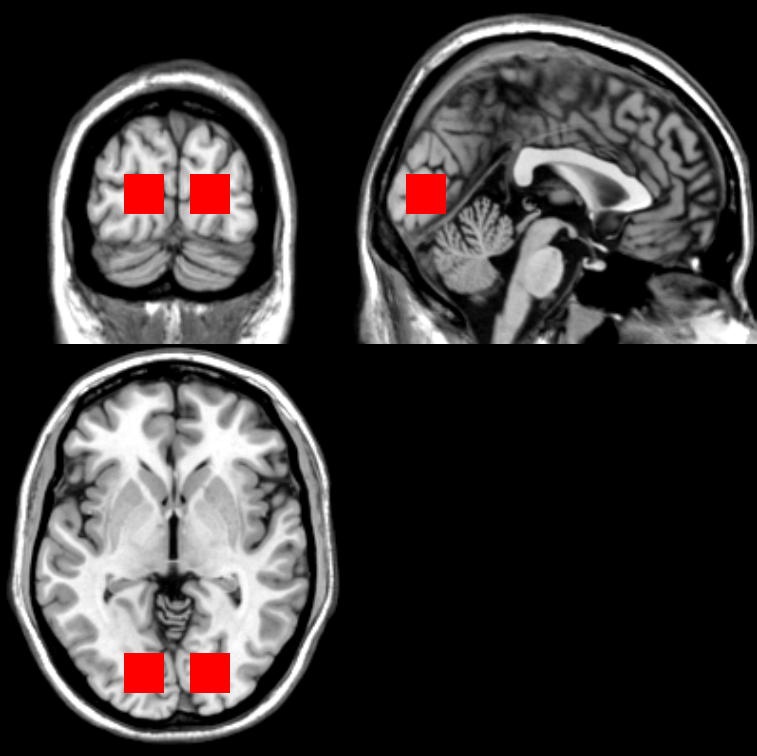

Supplement: Figure 1—source data 2. [file elife-71016-fig1-data2.zip › Figure 1 - Source Data 3/fig_1_data/mni_voxel.png]
